# Treatment of Conjunctivitis

**Published:** 1907-08-17

**Authors:** 


### Ophthalmology.

### TREATMENT OF CONJUNCTIVITIS.

### II. PURULENT AND GONORRHŒAL CONJUNCTIVITIS.

The "purulent variety" of conjunctivitis occurs most frequently as the "gonorrhœal ophthalmia" of adults, or as "ophthalmia neonatorum."

#### Prophylactic Treatment.

The prophylaxis of ophthalmia neonatorum should be a matter of routine in all midwifery practice. In suspected cases, the vagina should be well disinfected during labour. Immediately after birth the face and eyes of the infant should be carefully cleansed with lotio hydrarg. perchlor. (1 in 6,000), or with clean water, followed by the instillation into the eyes of gutt. argent. nit. 2 per cent. (Crédé's method). The importance of this cannot be over-emphasised.

So, in gonorrhœa, the practitioner should never forget to warn his patient of the grave dangers which may arise from the infection of the conjunctival sac with the gonococcus. Gonorrhœal ophthalmia of adults usually affects only one eye; on the other hand, ophthalmia neonatorum usually both. When one eye only is affected great care should be taken that the other does not become so; a Buller's shield, which consists of a square of thin mackintosh, with a watch glass let in, should be placed over the sound eye, and the margins carefully sealed down with gauze soaked in collodion, more especially the margin next the affected eye. At the outer margin a piece of small india-rubber tubing, with holes in it, should be inserted to secure good ventilation, otherwise the watch glass becomes obscured with moisture. The patient should sleep on the side of the affected eye to prevent the discharge passing over to the other side, and as far as possible he should be isolated from other persons.

#### Local Treatment.

In the first stage, when there is little discharge, antiseptic lotions only should be used. Boracic acid lotion alternating with lotio hydrarg. perchlor. 1 in 6,000, frequently applied, or a solution of permanganate of potash, freshly made at each application by the addition of the crystals to warm water in the proportion of 1 in 10,000.

The solutions are best given from a clean douche can, placed above the patient's head, with a sterilised glass nozzle at the end of the tube. It should be carefully observed that the edges of the nozzle are rounded off, and great care taken that the cornea is not injured in any way, as a septic ulcer, proceeding to sloughing of the cornea, may result. For this reason a syringe should never be used.

When there is much swelling and heat, iced compresses are beneficial at this stage, but they should be discontinued at the purulent stage. They are made of two or three layers of gauze or light lint of a little larger size than the eye, placed under a block of ice, a fresh piece being used each time. They may be applied for an hour or more, and with a frequency according to the benefit obtained.

If there is considerable tension of the lids on the globe, two or three leeches should be applied at the external canthus and free incisions with scissors made in the swollen conjunctiva. This gives much relief, but, in case of failure, divide the lids at the external canthus, cutting through the orbicularis muscle from without with a scalpel, encouraging bleeding at the time of operation. Repeated small doses of calomel tend to lessen the chemosis, and so the danger to the cornea from this source.

In the second or purulent stage, silver nitrate solution 10 grs. to 1 oz. should be painted on to the conjunctiva once a day in the way already described, the thorough irrigation of the conjunctival sac being continued. The swollen conjunctiva, when it overlaps the cornea, should be carefully pushed aside with a blunt probe and the cul-de-sac gently and well washed out—this is important.

The use of the mitigated silver stick, unless in skilled and experienced hands, may be attended with unhappy results, *e.g.* symblepharon (adhesion of lids to globe), and is not to be recommended. The use of a simple ointment, or ung. iodoformi, prevents the lids from adhering, and so facilitates a free drainage.

#### Membranous Conjunctivitis.

This form is characterised by the presence of a membrane on the conjunctiva. It consists of a fibrinous network, with leucocytes and the remains of necrotic epithelial cells in the meshes. If there is the least suspicion of the presence of the Klebs-Lœffler bacillus, antitoxin serum should be injected, with as little delay as possible, pending the definite diagnosis by bacteriological examination.

In the first stage of infiltration, cold compresses and antiseptic lotions should be used and a careful outlook kept for corneal complications. Soon the infiltration is thrown off, and a purulent discharge makes its appearance, more or less similar to that of purulent conjunctivitis, and to be treated in the same way with antiseptic lotions and caustic applications.

#### The Treatment of Corneal Complications.

The cornea should always be under careful observation. When surface abrasions or infiltrations are first seen, atropine is indicated, in addition to the local treatment, which is not in any way to be abated. The atropine lessens the tendency to iritis and diminishes pain. It may be most conveniently used in the form of drops 1 per cent., but when ulceration is present it should be prescribed as an ointment, the lubrication being an additional comfort. When the ulcer is septic, precipitated iodoform 60 grs. may be added to 1 oz. of ung. atropinæ with benefit.

When there is a corneal ulcer the eye is to be examined with greater care, for pressure on the globe may cause the ulcer to rupture. If the ulcer looks likely to perforate spontaneously, a linear incision should be made in its floor with a fine Graefe knife; the lowering of the tension by the escape of the aqueous promotes healing of the ulcer.

The iris usually becomes involved, the more so if perforation is spontaneous. Any prolapse of the iris now should not be cut off, for it is Nature's method of plugging up the perforation, and operative interference only opens up a direct tract for the incursion of the purulent infection into the deeper parts of the eye with disastrous results.

The little tag of iris involved in the scar of a small perforation towards the periphery of the cornea is called an "anterior synechia." If the small perforation is central the iris may not be involved at all, but the anterior capsule of the lens comes into apposition with the cornea. A proliferation of the intracapsular cells takes place at this situation, which, on the healing of the ulcer and the reformation of the anterior chamber, remains permanently as an "anterior polar capsular cataract."

When the perforation is large and more iris involved, the scar is dense and the iris adherent, so it is called "leucoma adherens"; should the scar be thin and bulge, a partial staphyloma of the cornea is the result.

